# Low-Salt or Salt-Free Dyeing of Cotton Fibers with Reactive Dyes using Liposomes as Dyeing/Level-Dyeing Promotors

**DOI:** 10.1038/s41598-018-31501-7

**Published:** 2018-08-29

**Authors:** Jidong Ru, Xueren Qian, Ying Wang

**Affiliations:** 1 College of Light Industry and Textile, Qiqihar University, Qiqihar, Heilongjiang Province, 161006 China; 2 Key Laboratory of Bio-based Material Science and Technology of Ministry of Education, Northeast Forestry University, Harbin, Heilongjiang Province, 150040 China; 3 College of Computer and Control Engineering, Qiqihar University, Qiqihar, Heilongjiang Province, 161006 China

## Abstract

The main aim of this investigation was to promote the dyeing and level-dyeing effect of reactive dyes on cotton-fiber dyeing by encapsulating reactive dyes in liposomes as an alternative to sodium chloride. The results obtained indicated that liposomes, especially cationic liposomes, have a remarkable level-dyeing promoting effect on cotton fibers, although the dyeing promoting effect was not as good as that of sodium chloride. The optimum dyeing and level-dyeing effects were achieved at a dye-fixing temperature of 85 °C, sodium carbonate concentration of 10 g/L and dye dosage of 2% (on the basis of oven-dry cotton fibers) when liposomes were used as the dyeing and level-dyeing promoters. The combination of cationic liposomes and sodium chloride can significantly promote both the dyeing and level-dyeing of cotton fibers. These results indicated the potential of cationic liposomes as novel dyeing and level-dyeing promoters or microencapsulated dye wall materials for reactive-dye dyeing applications.

## Introduction

Liposomes are also called lipid globules, and they are mainly composed of phospholipids^1^. Liposomes or phospholipid vesicles are amphoteric compounds containing both polar and nonpolar groups^2,3^. Liposomes are the assembling structures formed by surface-active biological lipids that are arranged with hydrophilic polar head groups exposed to the aqueous phase and hydrophobic fatty acid chains adhered together in the bilayer^4,5^. They form closed vesicles with a water core and an inner water domain embedded between the lipid bilayers^6^. Because liposomes are amphoteric compounds, they can wrap up various types of polar and nonpolar solutes. The polar solutes can be embedded within the aqueous compartment, while nonpolar solutes can be trapped between the bilayer^7^. Liposomes are nontoxic, harmless, and biodegradable^8,9^. Owing to the encapsulation and sustained-release abilities of liposomes, they are widely used in pharmaceuticals, textiles, detergents, foodstuffs, cosmetics and other fields^10^.

In textiles, liposome technology is mainly applied in the following six aspects: wool dyeing^11^, durable aroma finishing, textile protection^12^, parasiticide preparation, antibacterial drug preparation and phase-transition material preparation^13^. Liposomes have been examined as a means of delivering dyestuffs to fibers in a cost-effective and environmentally sensitive manner^14^. Compared with traditional retarding agents, liposomes can slowly release a microencapsulated dye to increase the retarding effect, making them a good alternative to commercial levelling agents^15^. Several papers have reported potential applications of liposomes for wool and wool/polyester mixture dyeing. Liposomes have been investigated as vehicles in wool dyeing with acid, disperse and metal-complex dyes, and wool and wool blend dyeing with liposomes resulted in better quality and energy savings and lower environmental impact^5,15–18^. The main advantages of using liposomes are an obvious reduction in the dyeing temperature (lower by approximately 10 °C compared with that of a traditional wool dyeing process); improved fiber quality, smoothness and mechanical properties of the dyed fibers; and an obvious reduction in the contamination load of the dye bath^6,19,20^. Recently, Marti *et al*. used an optimized mixture of commercial liposomes and cationic surfactant to improve the leveling effect. The presence of 1% liposomes during exhaustion at 85 °C improved the leveling effect of Irgalan Blue FBL, and the fastness properties of the fibers dyed with liposomes were also improved^21^. Research results from Sheveleva *et al*. showed that liposomes may be employed in the preparation of textile materials^22^.

The affinity of reactive dyes is generally low. Reactive dyes have a negative charge in water, and cellulose fibers are also electronegative in water. Therefore, electrostatic repulsion between cellulose fibers and anionic dyes can prevent reactive dyes from dyeing cellulose fibers. At present, a dyeing promoter is commonly used during reactive-dye dyeing. Inorganic salts (anhydrous sodium sulfate or sodium chloride) are the most commonly applied dyeing promoters. The cations of inorganic salts adsorb to the cellulose fiber surface, and the negative charge of the cellulose fiber is weakened. Thus, cellulose fibers can be dyed with anionic reactive dyes. However, inorganic salts are also bound resulting in severe salt pollution of the water body.

To solve the above problems, scholars have conducted the following work: (1) Low-salt or salt-free dyeing with reactive dyes such as the Sumifix Supra series of dyes from Japan Sumitomo Corporation has been developed. However, this kind of dye easily results in unlevel dyeing, which increases the dye dosage and dyeing cost. Therefore, changes in the dye structure cannot effectively solve the problem of high-salt dyeing of reactive dyes. (2) Cellulose fibers have been cationically modified. However, cationic modification affects the level-dyeing property of cellulose fibers, and the most important disadvantage of this modification is that it is difficult to use for large-scale application^23^. (3) Low-salt or salt-free dyeing auxiliaries have been developed. However, the pretreatment of cellulose fibers with an auxiliary is a complex production process that also leads to low friction fastness and high stiffness of the dyed fabrics. Meanwhile, low-salt dyeing auxiliaries are prone to yellow in high temperature and are unfit dyes for light-color fabrics. (4) Substitutes for inorganic salts have been developed. For example, ethylenediamine tetraacetic acid was studied as an alternative to inorganic salts^24^. In summary, the above efforts still have some problems, although the dosage of inorganic salt required was reduced by varying degrees. For that reason, nontoxic, harmless and biodegradable liposomes were synthesized by using soybean lecithin and stearamide as raw materials, and were used to promote the dyeing of cotton fibers with reactive dyes by encapsulating reactive dyes in liposomes as an alternative to sodium chloride. The environmental pollution caused by the use of traditional dyeing promoter can be reduced. In addition, liposomes can also improve the level-dyeing effect of reactive dyes dyeing compared with sodium chloride because liposomes can encapsulate reactive dyes in the lipid bilayer and have long-term and sustained-release effect.

In this work, neutral nanoliposomes (abbreviated NL) and cationic nanoliposomes (abbreviated CL) were applied as novel dyeing and level-dyeing promoters to enhance the dyeing effect of reactive dyes on cotton-fiber dyeing by encapsulating reactive dyes in nanoliposomes as an alternative to inorganic salt. The morphology, particle size and zeta potential of CL and NL were studied; the dyeing effects of three kinds of dyeing and level-dyeing promoters (CL, NL and NaCl) were compared; and the dyeing-promotion and level-dyeing mechanisms of NL and CL on reactive dyes were analyzed. The effects of the dye-fixing temperature, Na_2_CO_3_ concentration and dye dosage on the dyeing of cotton fibers were studied to determine the optimal dyeing conditions, and a combination of NL/CL and NaCl was used to further improve the dyeing-promotion and level-dyeing effects.

## Materials and Methods

### Materials

Commercial soybean lecithin, comprising 28 mol% phosphatidylcholine, 16 mol% phosphatidylethanolamine, 14 mol% phosphatidylinositol, and 4 mol% phosphatidic acid as the main ingredients, was provided by Merya’s Lecithin Co., Ltd. (Beijing, China). Stearamide was purchased from Sigma Chemical Co. (St. Louis, MO, USA). Bleached cotton fabric (32 cm × 68 cm) was obtained from Keshan Jinding Linen Textile Co., Ltd. (Qiqihar, China). Dyes (reactive red 3BS, reactive yellow 3RS and reactive blue FBN) were produced by Everlight Chemical Industrial Corporation (Taiwan). All other reagents were analytically pure and used as received.

#### Preparation of NL and CL

Before the preparation of NL and CL, commercial soybean lecithin was purified for 20 min at 36 °C under 25 MPa by a supercritical carbon dioxide extractor (Hua’an Supercritical Fluid Extraction Co., Ltd., Nantong, China). NL and CL were prepared following a thin-film hydration method. Briefly, soybean lecithin (or soybean lecithin and stearamide) was entirely dissolved in chloroform and sonicated for 5 min at 25 °C. Chloroform at 40 °C (i.e., the water-bath temperature) and 50 rpm was entirely distilled. Finally, a thin lipid layer was formed on the sides of the round-bottom flask. The round-bottom flask was dried overnight in a vacuum drying oven to completely remove residual chloroform. Then, deionized water was added to the bottom of the round-bottom flask, and the thin-film lipid was adequately hydrated and completely separated from the round-bottom flask through high-speed stirring^25,26^. Nanoliposome emulsions were sonicated again for 5 min at 25 °C. Finally, the nanoliposome emulsion was treated 5 times under 60 MPa using a high-pressure homogenizer (Shanghai Precise Machinery Equipment Co., Ltd., China).

#### Dyeing of Cotton Fabric

Dyeing experiments were performed in a thermostat water bath with a dye-bath ratio of 1:30. Only one piece of cotton fabric (6 g) was used in each dye bath. The dosage of the dye (reactive red 3BS) was 2% (on the basis of oven-dry cotton fibers), the concentration of Na_2_CO_3_ was 10 g/L, and the concentration of both NL and CL was 84 mmol/L (NL and CL concentrations given in the Experimental section were optimized for the system). The dyeing temperature was kept at 60 °C for 30 min, increased to 85 °C at a gradient of 1 °C/min and kept at 85 °C for 40 min unless specified otherwise.

### Characterization

#### Morphology

The morphologies of NL and CL were obtained using an H-7650 transmission electron microscope (Hitachi, Japan). A colloidal solution of NL and CL was appropriately diluted, and then, a special copper net covered with a carbon film was dipped into the solution. Next, NL and CL were negatively stained with a phosphotungstic acid solution (20 g/L), and finally, the morphology and size of NL and CL were observed and estimated.

#### Zeta potential and particle size

NL and CL colloidal solutions were diluted to 1.5 mL with a phosphate buffer solution (pH 7.6), and the zeta potential and particle size of NL and CL were measured on a Zetasizer Nano ZS90 (Malvin Instrument, UK).

#### Testing

*K/S* was measured using a SF600X Datacolor Chroma Spectrometer (Datacolor, US) at the maximum absorption wavelength. A higher *K/S* value represented a darker fabric, and a lower *K/S* represents a lighter fabric.

Ten points on the dyed pure cotton samples were taken to measure the *K/S*, and then, the unlevel-dyeing property of the dyed fabric (*U*) was calculated by the following formula:

1 $$U=\sqrt{\frac{\sum \,{[\frac{{(K/S)}_{i}}{\overline{(K/S)}}-1]}^{2}}{n}}$$

where (*K/S*)_*i*_ is the *K/S* of the *i* point on the sample, and *n* is the number of measurements. A lower *U* value represents a better level-dyeing property of the dyed fabric^27^.

Soaping fastness was determined according to the Chinese National Standard GB/T 3921–2008, and friction fastness was determined according to the Chinese National Standard GB/T 3920–2008.

## Results and Discussion

### NL and CL characterization

**Figure 1:**
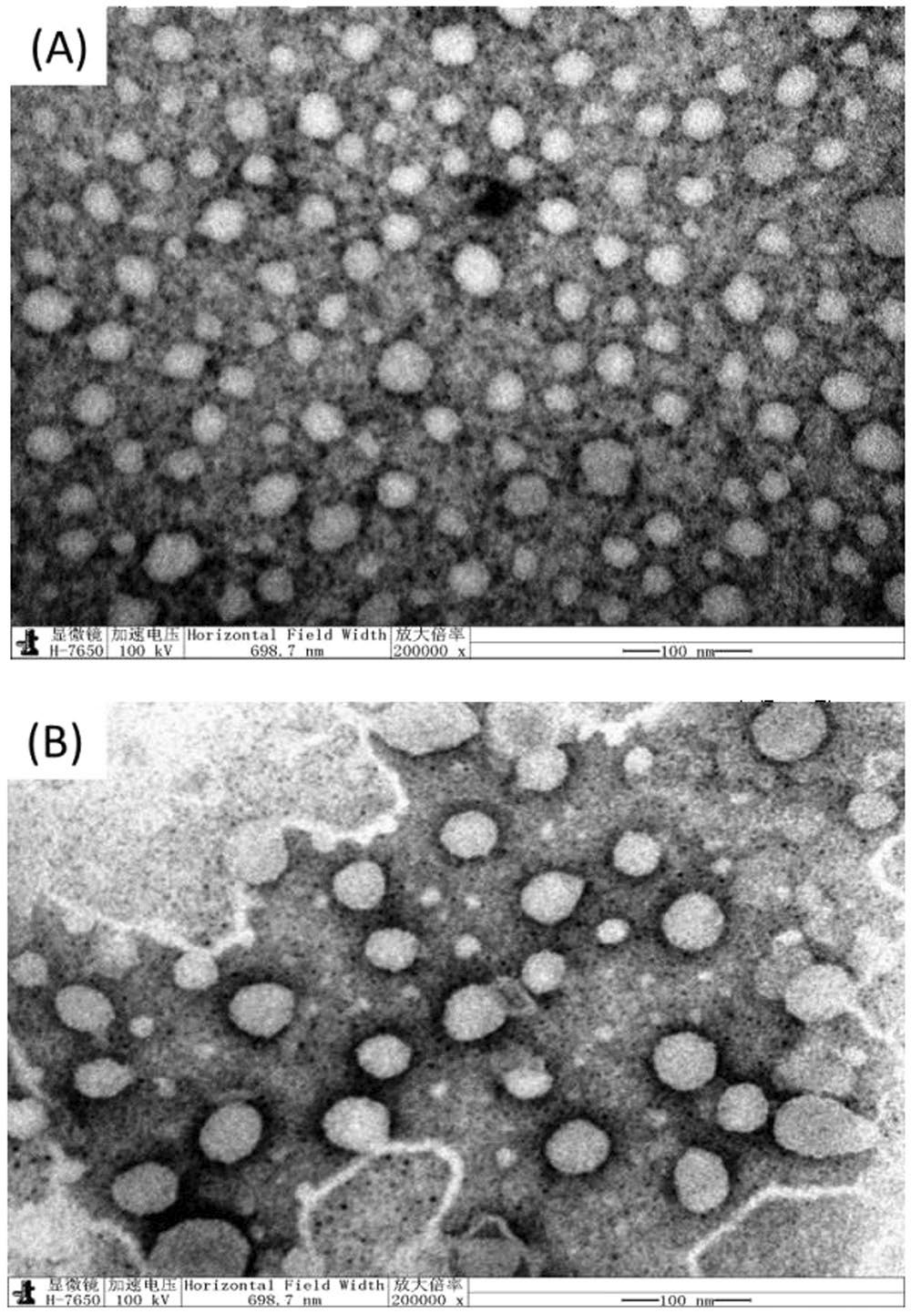
TEM images of NL **(A)** and CL **(B)**.

The NL and CL were prepared and characterized at the beginning of this work. The transmission electron microscopy (TEM) images of NL and CL are shown in Fig. 1. NL and CL particles were basically spherical, and their particle sizes were mainly uniform. On account of the addition of stearamide, the size of CL (approximately 30 nm) was greater than that of NL (approximately 20 nm). Compared with that of NL, the dispersity of CL was also better due to the repulsive interactions between CL particles. The particle size and zeta potential of NL and CL were determined using a Zetasizer Nano ZS90, and the results are shown in Table 1. The average particle size of NL was 30.47 nm, whereas the average particle size of CL was 41.71 nm due to the addition of stearamide. The inconsistencies in the NL and CL particle sizes in Fig. 1 and Table 1 might be caused by the difference in the measuring principles of the two methods. The particle sizes from the Zetasizer Nano ZS90 (Table 1) data are the mean values of all nanoliposome particles, while those from the TEM observation (Fig. 1) are estimated values of some nanoliposome particles. We think that the nanoliposome particle sizes in Table 1 are more accurate. The zeta potential values of NL and CL (Table 1) showed that the potential of NL was almost electrically neutral (−1.69 mV), yet the potential of CL was obviously positive (+27.8 mV), which could be attributed to the addition of stearamide.

**Table 1:** Particle sizes and zeta potentials of NL and CL.

Liposome type	Particle size (nm)	Zeta potential (mV)	Zeta deviation (mV)
NL	30.47	−1.69	8.04
CL	41.71	+27.8	5.62

### NaCl, NL and CL-mediated reactive-dye dyeing

The dyeing effect on cellulose fibers using NaCl, NL and CL as dyeing and level-dyeing promoters under the optimal conditions was studied, and the results are shown in Table 2. Comparing the dyeing-promotion properties, the results clearly show that the order for high to low *K/S* values was as follows: NaCl, CL, NL, and without any dyeing and level-dyeing promoters. For the level-dyeing promotion, the enhancement of level-dyeing from high to low was as follows: CL, NL, NaCl, and without any dyeing and level-dyeing promoters. The K/S of the fabric without any dyeing and level-dyeing promoters was very low, and the fabric was merely stained. Liposomes, especially CL, had a great level-dyeing promoting effect on cotton fibers, although the dyeing promoting effect was not as good as that of NaCl. Therefore, NL and CL obviously perform the level-dyeing promoting role in the dyeing process of reactive dyes. Table 2 also shows that only light-color fabrics can be dyed using NL and CL as dyeing and level-dyeing promoters. NaCl must be used as a dyeing promoter to obtain dark-color fabrics. Liposomes, especially CL, have remarkable level-dyeing promoting effects on cotton fibers compared with NaCl and without any dyeing and level-dyeing promoters. Clearly, the soaping fastness and friction fastness were not much different, although different dyeing and level-dyeing promoters were used in the reactive-dye dyeing process.

**Table 2:** Dyeing effect of cotton fabrics with three dyeing and level-dyeing promoters.

Evaluating indicator		Reactive red 3BS				Reactive yellow 3RS				Reactive blue FBN			
		A	B	C	D	A	B	C	D	A	B	C	D
K/S		0.55	4.16	6.21	10.08	0.32	1.16	1.87	3.35	0.63	3.57	5.44	7.92
level-dyeing property (10^−2^)		3.4	1.5	1.0	2.9	3.1	1.6	0.9	2.7	3.4	1.9	1.3	3.6
soaping fastness	fade/grade	4–5	5	5	5	4–5	5	5	5	4–5	5	5	5
	silk staining/grade	4–5	5	5	5	4–5	5	5	5	4–5	4–5	4–5	4–5
	cotton staining/grade	4–5	5	5	5	4–5	5	5	5	4–5	4–5	4–5	4–5
dry friction/grade		4	4–5	4–5	4–5	4	4–5	4–5	4–5	3–4	4–5	4–5	4–5
wet friction/grade		3–4	4	4	4	3–4	4–5	4–5	4–5	3	4	4–5	4–5

Reaction conditions: A-without any dyeing and level-dyeing promoter; B-NL 84 mmol/L; C-CL 84 mmol/L; D-NaCl 60 g/L.

Reactive dyes have a negative charge in water, and cellulose fibers are electronegative in water. Therefore, the electrostatic repulsion between cellulose fibers and anionic reactive dyes can prevent reactive dyes from dyeing cellulose fibers. At present, a dyeing promoter is commonly used during reactive-dye dyeing.

NaCl acts as a dyeing promoter in traditional cotton fabric dyeing with reactive dyes. The Na^+^ ions of NaCl are adsorbed by cellulose fibers due to electrostatic attraction between Na^+^ and the cellulose fibers, and the negative charge of the cellulose fibers is weakened. Thus, the electrostatic repulsion between cellulose fibers and anionic reactive dyes is reduced, allowing cellulose fibers to be dyed with anionic reactive dyes. However, the use of a large amount of NaCl results in severe salt pollution of the water body.

As far as the intermolecular force between two molecules or ions is concerned, Coulomb force (electrostatic force) is much larger than van der Waals force. When cellulose fibers with negative charges are dyed with reactive dyes, the large Coulomb repulsion between dyes and fibers makes the dyes could not approach the fiber surface. NL and CL can encapsulate and transport reactive dyes into fiber surface, and the released reactive dyes are quickly adsorbed by cellulose fibers due to van der Waals force. Therefore, NL and CL can act as dyeing promoter during the dyeing of cellulose fibers. The larger the thickness of vesicle bilayer, the smaller the electrostatic repulsion of fiber and dye, but the harder the release of the dye from the vesicle.

**Figure 2:**
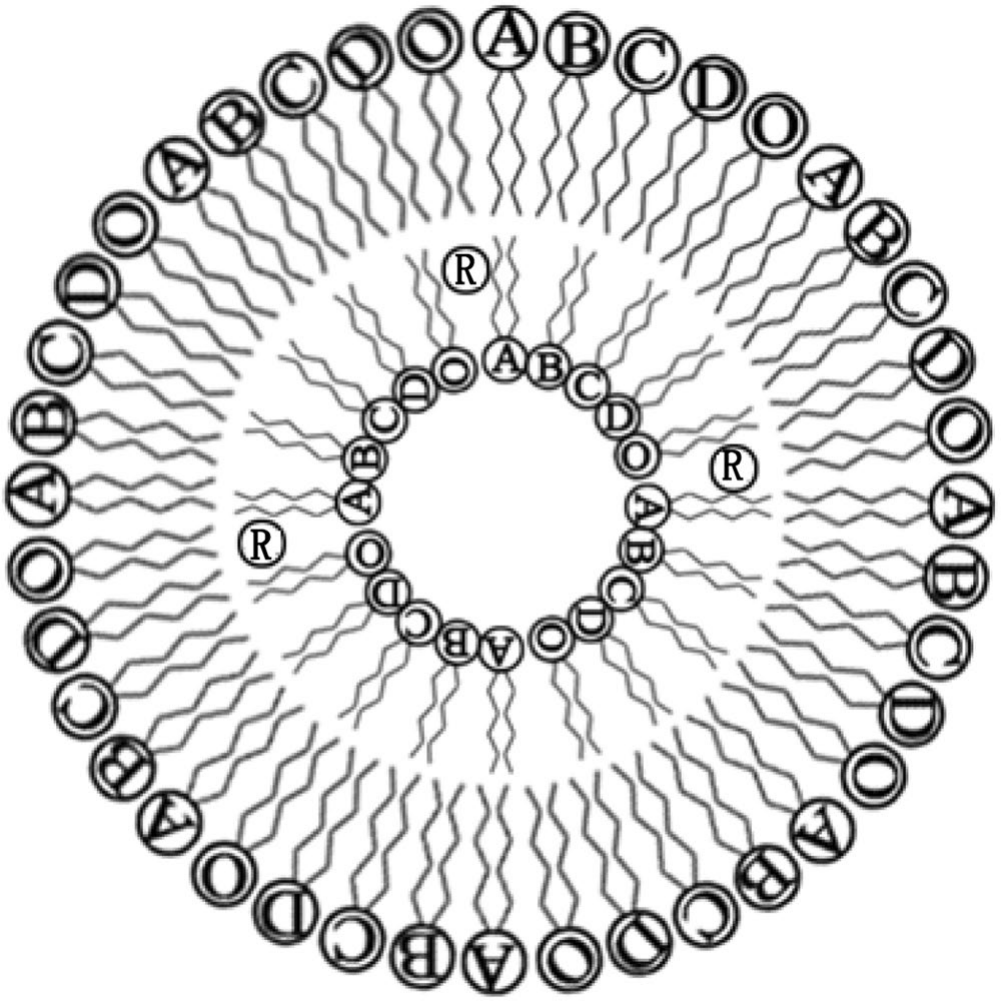
Proposed mechanism for the interaction of reactive dye (R) with NL and NL ingredients; **(A)** phosphatidylcholine, **(B)** phosphatidylethanolamine, **(C)** phosphatidylinositol, **(D)** phosphatidic acid, and **(O)** other ingredients.

The dyeing-promotion and level-dyeing mechanisms of NL in reactive-dye dyeing of cellulose fibers are presented (Fig. 2). The dyeing and level-dyeing promotion mechanisms of NL are entirely different from those of NaCl. NL has a lipid bilayer structure, which can extensively encapsulate various polar and nonpolar solutes. At lower temperatures, the dye is in a less hydrated environment in lecithin liposomes, which corresponds to a location in the liposome membrane, probably nears the polar head groups. With increasing temperature, the dye relocates to a more hydrated environment, and is released from the liposome membrane to the aqueous solution^28^ (Fig. 2). Cellulose fibers and reactive dyes are all negatively charged in water, and the electrostatic repulsion between cellulose fibers and reactive dyes can prevent reactive dyes from dyeing cellulose fibers. After reactive dyes are encapsulated by NL, reactive dyes are converted into microcapsule dyes with electrically neutral NL as the wall materials. There are no electrostatic repulsion between cellulose and NL, so reactive dyes can be transported to the surface of cellulose fibers by molecular motion of the electroneutral NL. With the increase of temperature, the diffusion activation energy and the uptake of reactive dyes are also increased. The reactive dyes are slowly released from the NL membrane to reach the surface of the cellulose fibers, and covalently react with the hydroxyl groups of cellulose fibers under alkaline conditions. In this way, the reactive dyes are firmly fixed to the cellulose fibers. NL can play a dyeing promoter role in the dyeing process of reactive dyes. In addition, if the dyeing rate is too fast, the unlevel-dyeing of cellulose fibers would occur. Liposomes have a slow-release property, the dyes encapsulated in NL are slowly released from NL, thereby avoiding the unlevel-dyeing phenomenon caused by fast dyeing. Therefore, the level-dyeing property of the dyed fabric using NL as a dyeing and level-dyeing promoter was obviously better than that of the one dyed using NaCl.

**Figure 3:**
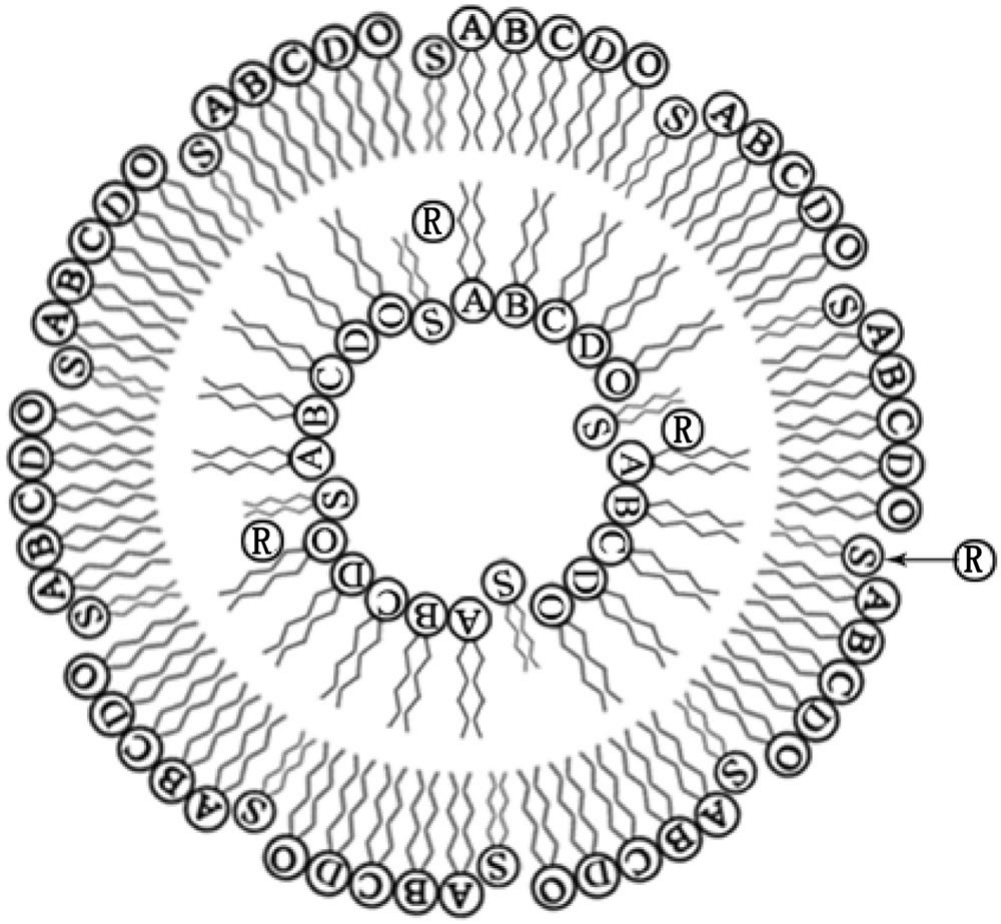
Proposed mechanism for the interaction of reactive dye (R) with CL and CL ingredients: **(A)** phosphatidylcholine, **(B)** phosphatidylethanolamine, **(C)** phosphatidylinositol, **(D)** phosphatidic acid, (O) other ingredients, and (S) stearamide.

A CL formulation with soybean phospholipids and stearamide was used to further improve the dyeing-promotion and level-dyeing properties of reactive dyes, and the dyeing-promotion and level-dyeing mechanisms of CL in reactive-dye dyeing of cellulose fibers are also presented. Figure 3 shows a graphical representation of the interaction of reactive dyes with CL components. This interaction leads us to expect a better improvement in the dyeing-promotion and level-dyeing properties of reactive dyes. Reactive dyes encapsulated by CL can be more easily adsorbed on cellulose fibers due to the electrostatic attraction between CL and cellulose fibers compared with that of NL. At the same time, the negative charges of cellulose fibers decrease due to adsorption of positively charged CL, and some reactive dyes that are unencapsulated by CL in the dyeing solution will continue to be adsorbed on the cellulose fibers because the negative charge of the cellulose fibers is reduced. That is, due to the double dyeing-promotion effect of CL, the *K/S* values of the fabrics dyed using CL as the dyeing and level-dyeing promoter were better than those of the fabrics using NL. Similarly, reactive dyes encapsulated in CL can also be electrostatically attracted by CL when they are released from CL (Fig. 3), so CL can significantly retard the release of encapsulated reactive dyes^28^. Therefore, CL has a better level-dyeing promoting effect than NL and NaCl.

### Influence of the dye-fixing temperature on dyeing

**Figure 4:**
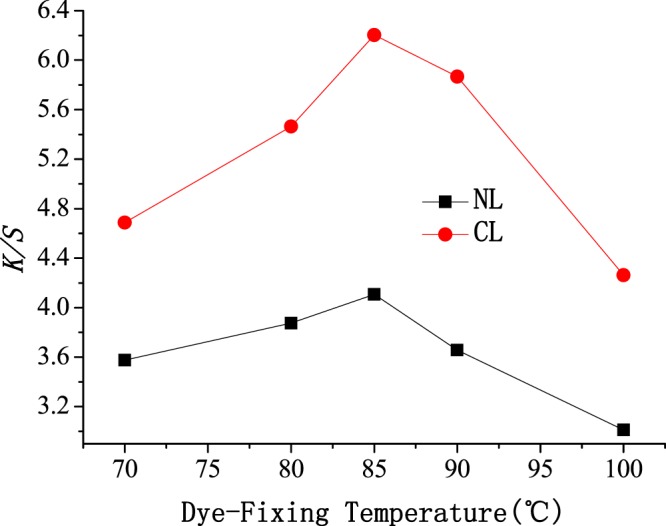
Effect of dye-fixing temperature on *K/S* of cotton fabric.

The influence of the dye-fixing temperature on dyeing was investigated using NL and CL as dyeing and level-dyeing promoters, and the results are shown in Fig. 4. The *K/S* values of the cotton fabrics dyed using NL and CL as the dyeing and level-dyeing promoters had a similar trend as the dye-fixing temperature increased from 70 to 100 °C. The *K/S* values all first increased and then decreased, and the optimum *K/S* was achieved at 85 °C. Increasing the dye-fixing temperature can improve the diffusion activation energy and dyeing rate of reactive dyes. Therefore, an appropriate increase in the dye-fixing temperature will improve the *K/S*. However, the *K/S* values of the dyed fabric decreased when the dye-fixing temperature exceeded 85 °C. Increasing the dye-fixing temperature not only increases the reaction rate of reactive dyes with cellulose fibers but also increases the hydrolysis rate of the reactive dyes. The increase in the reactive-dye hydrolysis rate might be greater than the increase in the reactive-dye dyeing rate upon increasing the dye-fixing temperature from 85 °C to 100 °C. Another explanation might be that the lipid bilayer structure of NL and CL was destroyed at high temperature. Then, NL and CL become dispersed phospholipids, and the dispersed phospholipids can be deposited on the surface of the cotton fabric, which would hinder continuous dyeing of the cotton fibers.

### Influence of the Na_2_CO_3_ concentration on dyeing

**Figure 5:**
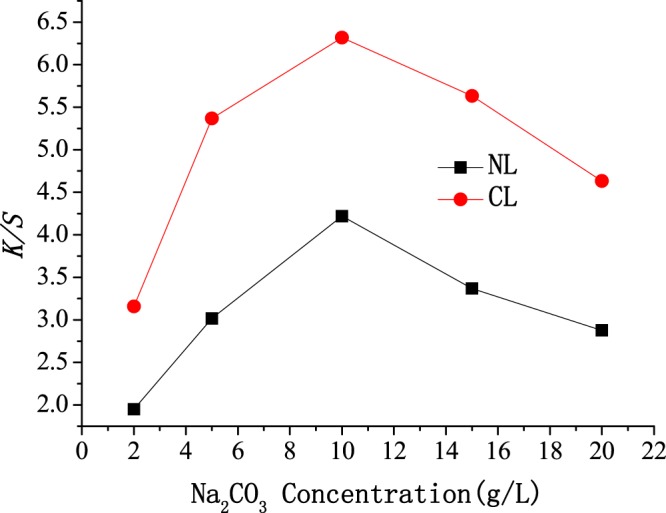
Effect of Na_2_CO_3_ concentration on *K/S* of cotton fabric.

To understand the influence of the Na_2_CO_3_ concentration on dyeing, the relationship between *K/S* and the Na_2_CO_3_ concentration was also studied using NL and CL as dyeing and level-dyeing promoters. As observed in Fig. 5, the influence of the Na_2_CO_3_ concentration on K/S is very significant. *K/S* first increased and then decreased as the Na_2_CO_3_ concentration increased from 2 to 20 g/L, and the optimum *K/S* was achieved at 10 g/L. Reactive-dye dyeing can be divided into two stages. In the first stage, reactive dyes are adsorbed on cellulose fibers as much as possible in the near-neutral dyeing bath, and adsorption equilibrium of reactive dyes on cellulose fibers is achieved. In the second stage, the reactive dyes adsorbed on cellulose fibers covalently react with the hydroxyl groups on the cellulose fibers, i.e., the fixation reaction of reactive dyes. Increasing the pH of the dyeing bath can improve the reactivity of the hydroxyl groups on the cellulose fibers; therefore, the fixation reaction rate of reactive dyes can be improved. Sodium carbonate can improve the pH of the dyeing bath; i.e., the pH of the dyeing bath increases by adding Na_2_CO_3_. The covalent reaction between the hydroxyl groups on the cellulose fibers and the reactive dyes is gradually accelerated by increasing the pH, and the dye-fixing reaction is complete. Meanwhile, the adsorption equilibrium of reactive dyes is also destroyed as the dye-fixing reaction proceeds, and some reactive dyes unencapsulated from NL and CL will continue to attach to cellulose fibers. The *K/S* first increased and then decreased with the increasing pH. This result might be explained by the increase in pH improving the dye-fixing reaction rate and the ionization degree of hydroxyl groups on cellulose fibers. The negative charges on cellulose fibers increase with the improving degree of hydroxyl-group ionization. However, at a much higher pH, electrostatic repulsions between reactive dyes unencapsulated from NL and CL and the cellulose fibers increase, which hinders the adsorption of reactive dyes by cellulose fibers, so the *K/S* decreases. The hydrolysis of reactive dyes at a much higher pH is accelerated, which might be another reason why the *K/S* decreased.

### Influence of dye dosage on dyeing

**Figure 6:**
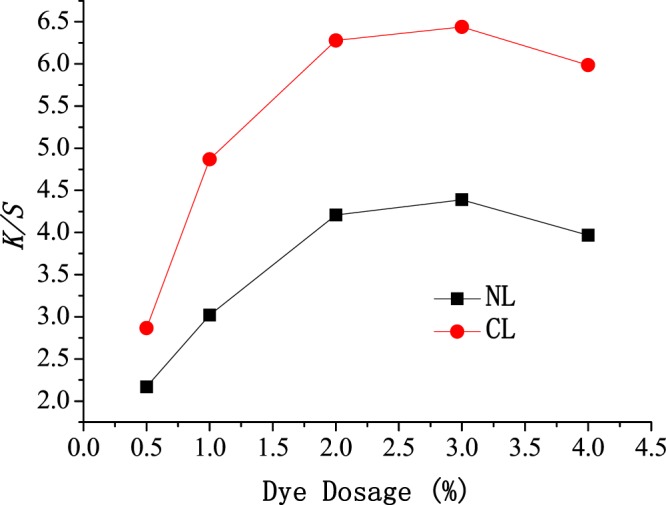
Effect of dye dosage on *K/S* of cotton fabric.

The dye dosage is one of the most important factors affecting dyeing. To understand the influence of the dye dosage on dyeing, the relationship between the dye dosage and *K/S* was also studied using NL and CL as the dyeing and level-dyeing promoters, and the results are shown in Fig. 6. The *K/S* first increased and then decreased with the increasing dye dosage, but the *K/S* only slightly increased as the dye dosage increased from 2% to 3% (on the basis of oven-dry cotton fibers). Thus, the optimum *K/S* was achieved at 2% considering dye savings. The dyes encapsulated in NL and CL also increased with the increasing dye dosage. More reactive dyes encapsulated in NL and CL were released and attached to the fibers, so the *K/S* increased with the increasing dye dosage. However, reactive dyes tend to aggregate when the dye dosage is too high, and aggregated dyes cannot be adsorbed on cellulose fibers. Similarly, aggregated dyes cannot be encapsulated by NL and CL. That is, the aggregated dyes cannot be transferred onto cellulose fibers by NL and CL. Therefore, the *K/S* decreased when the dye dosage was too high.

### Dyeing by a combination of NaCl and NL/CL

To further improve the dyeing and level-dyeing effects, a combination of NaCl and NL/CL was used in dyeing. Under the optimum dyeing conditions, the *K/S* and level-dyeing properties were investigated, and the results are shown in Table 3. The dosage of NL/CL was constant, and the concentration of NaCl was increased from 5 g/L to 30 g/L. The *K/S* of dyeing using both 84 mmol/L NL and 30 g/L NaCl as the dyeing and level-dyeing promoters, respectively, was equal to that using only 60 g/L NaCl as the dyeing promoter, and the *K/S* of dyeing using both 84 mmol/L CL and 20 g/L NaCl was equal to that using only 60 g/L NaCl. In other words, to obtain a sample with the same color, dyeing with a combination of NL and NaCl can reduce the NaCl needed by half compared with that needed using only NaCl as the dyeing promoter. Dyeing with a combination of CL and NaCl can reduce the amount of NaCl by two-thirds compared with that using only NaCl as the dyeing promoter. The dyeing-promotion effect of CL was better than that of NL. In addition, the level-dyeing property of dyeing using both NL/CL and NaCl as the dyeing and level-dyeing promoters was much better than that using only NaCl, and the level-dyeing property of dyeing with a combination of CL and NaCl was better than that of dyeing with a combination of NL and NaCl.

**Table 3:** Results of dyeing by combination of NaCl and NL/CL.

Dyeing promoter	Reactive red 3BS		Reactive yellow 3RS		Reactive blue FBN	
	*K/S*	level-dyeing property (10^−2^)	*K/S*	level-dyeing property (10^−2^)	*K/S*	level-dyeing property (10^−2^)
84 mmol/L NL + 5 g/L NaCl	5.45	1.4	1.73	1.3	4.21	1.9
84 mmol/L NL + 10 g/L NaCl	6.54	1.6	2.07	1.5	5.10	2.1
84 mmol/L NL + 20 g/L NaCl	8.47	1.8	2.82	1.6	6.62	2.2
84 mmol/L NL + 30 g/L NaCl	9.87	1.9	3.40	1.6	7.92	2.5
84 mmol/L CL + 5 g/L NaCl	7.57	1.0	2.21	0.9	5.98	1.4
84 mmol/L CL + 10 g/L NaCl	8.54	0.9	2.84	1.1	6.79	1.5
84 mmol/L CL + 20 g/L NaCl	10.04	1.0	3.39	1.0	7.97	1.7
84 mmol/L CL + 30 g/L NaCl	10.11	1.2	3.41	1.1	8.10	1.9
60 g/L NaCl	10.08	3.0	3.41	2.8	8.05	3.7

## Conclusions

Cotton fibers were dyed by reactive dyes encapsulated in liposomes, and the optimum dyeing effect was achieved at a dye-fixing temperature of 85 °C, Na_2_CO_3_ concentration of 10 g/L and dye dosage of 2% (on the basis of oven-dry cotton fibers). In regard to the dyeing-promotion property, the sequence of *K/S* ordered from high to low was as follows: NaCl, CL, and NL. However, for the level-dyeing promotion property, the sequence of level-dyeing property from high to low was CL, NL, and NaCl. Only light-color fabrics can be dyed using NL and CL as dyeing and level-dyeing promoters. NL/CL and NaCl must be combined to obtain dark-color fabrics. Dyeing with a combination of NL and NaCl can reduce the amount of NaCl by half, and dyeing with a combination of CL and NaCl can reduce the amount of NaCl by two-thirds. These results indicate the potential of CL as a novel dyeing and level-dyeing promoter or microencapsulation dye wall material for reactive-dye dyeing applications. The dyeing and level-dyeing promotion mechanisms of NaCl, NL and CL were proposed.
